# Statistical Optimization of Medium for Pullulan Production by *Aureobasidium pullulans* NCPS2016 Using Fructose and Soybean Meal Hydrolysates

**DOI:** 10.3390/molecules23061334

**Published:** 2018-06-01

**Authors:** Jinyu Yang, Yanhao Zhang, Shuangzhi Zhao, Qingxin Zhou, Xue Xin, Leilei Chen

**Affiliations:** 1 Institute of Agro-Food Science and Technology, Shandong Academy of Agricultural Sciences, Key Laboratory of Agro-Products Processing Technology of Shandong Province, Key Laboratory of Novel Food Resources Processing, Ministry of Agriculture, 202 Gongye North Road, Jinan 250100, China; yangjinyu86@163.com (J.Y.); zhang_8497@163.com (Y.Z.); 13589111848@163.com (S.Z.); zhouqx0211@163.com (Q.Z.); jasmine811201@126.com (X.X.); 2 College of Life Science, Shandong Normal University, Jinan 250014, China

**Keywords:** *Aureobasidium pullulans*, fructose, pullulan, response surface methodology, optimization

## Abstract

Pullulan, with its excellent characteristics of film-forming, water solubility, and biodegradability, is attracting more and more attention in agricultural products preservation. However, high pullulan production cost largely restricts its widely application due to its low production. In order to improve pullulan production by *Aureobasidium pullulans* NCPS2016, the medium was optimized using single factor experiment and response surface methodology. Based on the single factor experiments, the contents of soybean meal hydrolysates (SMHs), (NH_4_)_2_SO_4_, and K_2_HPO_4_·3H_2_O were considered to be main factors influencing the extracellular polysaccharide (EPS) production, and were further optimized by Box–Behnken design. The optimal content of SMHs of 7.71 g/L, (NH_4_)_2_SO_4_ of 0.35 g/L, and K_2_HPO_4_·3H_2_O of 8.83 g/L were defined. Finally, EPS production of 59.8 g/L was obtained, 39% higher in comparison with the production in the basal medium. The purified EPS produced by NCPS2016 was confirmed to be pullulan. This is the first time fructose is reported to be the optimal carbon source for pullulan production by *Aureobasidium pullulans*, which is of great significance for the further study of the mechanism of the synthesis of pullulan by NCPS2016. Also, the results here have laid a foundation for reducing the industrial production cost of pullulan.

## 1. Introduction

Pullulan is a kind of extracellular water-soluble polysaccharide secreted by *Aureobasidium* spp., which is a linear macromolecule linked by α-(1,6) glycosidic linkages of maltotriose repeating units [1,2]. The unique structure of pullulan makes it highly water soluble and biodegradable, so that it is a functional biological macromolecule with great development values and application prospects [3]. Pullulan has been widely applied in food processing and preservation in recent years [4], and it is considered as a new kind of material for food preservation because of its fine film-forming property [5]. Pullulan coating has been applied to protect perishable agricultural products especially susceptible to mechanical injury including vegetables, sea foods, and fruits. Pullulan coating incorporated ethanol extracts of meadowsweet flower could decrease the intensity of the decay of the red peppers initiated by *Rhizopus arrhizus*, which is 5-fold lower than that of the uncoated peppers at 24 °C for 5 days. During the storage, the pullulan coated peppers show less changes in color, weight loss, and total soluble solid content after 30 days at 6 °C than that of uncoated peppers, owing to a thin and well-attached film on the surface of the coated peppers [6]. Pullulan mixed with 0.05% reducing glutathione, 0.5% citric acid, and 0.2% potassium sorbate is used for preservation of razor clam *Sinonovacula constricta* meats, which could extend the shelf life to 10 days during refrigerated storage (∼2 °C) [7]. Besides, pullulan coating could extend the shelf life of highbush blueberry to 14 days at 16 °C, and at refrigerator temperature (4 °C), the storage period could be extended to 28 days because pullulan coating acts as a barrier that decreases significantly the respiration rate [8]. In the biomedical fields, pullulan has been used as a carrier for gene delivery or target drug therapy due to the presence of the hydroxyl groups which could be derivatized in varied forms. Pullulan modified by folate-low-molecular-weight polyethyleneimine could be used as gene carrier candidate for delivery of folate receptor (FR)-mediated gene/short interfering RNA (siRNA) into specific FR-overexpressing cancer cells [9], and nanoparticles including charge reversible pullulan-based shell packing poly(β-aminoester)/poly(lactic-co-glycolicacid) could be developed as a carrier of paclitaxel and combretastatin A4, to treat hepatocellular carcinoma [10]. In addition, pullulan has great application value in the cosmetics industry, because of its fine film-forming property, transparency, moisture absorption, water solubility, and stickiness. Since pullulan is non-toxic and non-irritating to human body, it is suitable for use in lotions, eye care products, shampoos, and toothpastes [11]. However, that the production cost of pullulan is relatively high greatly limits its extensive applications, especially in the application of the preservation of agricultural products. At present, it is considered that the screening of high-production strains of pullulan and the optimization of the fermentation conditions of available strains to improve the production of pullulan are effective methods to solve this problem.

To date, *Aureobasidium* spp. contains seven species: *Aureobasidium pullulans*, *Aureobasidium leucospermi*, *Aureobasidium proteae*, *Aureobasidium thailandense*, *Aureobasidium melanogenum*, *Aureobasidium subglaciale* and *Aureobasidium nambiae* [2,12], of which *A. pullulans* is considered to be excellent species for pullulan production [10,13], while the different strains of *A. pullulans* show different abilities to produce pullulan [14,15,16]. *A. pullulans* could utilize a variety of carbon sources to synthesize pullulan. Most research showed that sucrose is the optimal carbon source for pullulan synthesis [17,18,19], while xylose and lactose are not conducive to the synthesis of pullulan [17]. It has also been pointed out that *A. pullulans* can efficiently utilize glucose to synthesize pullulan [14,20]. The carbon source is an important factor to influence the production of pullulan, and in addition, nitrogen source also plays an important role in the synthesis of pullulan. In most studies, a combination of yeast extract and ammonium sulfate was used as the nitrogen source for the production of pullulan [18,19,21]. In order to reduce the production cost, some complex nitrogen sources, such as corn pulp, oil-removed rapeseed cakes, etc. have also been used as nitrogen sources to produce pullulan. Studies have indicated that these complex nitrogen sources are more beneficial for improving the production of pullulan than inorganic nitrogen sources [22,23].

In this study, a pullulan-producing strain was identified by the rRNA internal transcribed spacers sequence (ITS), and named by *A. pullulans* NCPS2016. NCPS2016 showed higher production using fructose to synthesize pullulan than using glucose or sucrose. In order to improve the pullulan production and reduce the pullulan production cost, the medium for pullulan production was optimized by single factor experiment and response surface methodology (RSM) using fructose as carbon source and soybean meal hydrolysates (SMHs) as nitrogen source. Finally, the production of pullulan by NCPS2016 increased by 39%, which is of important guiding significance for future industrial production of pullulan.

## 2. Results and Discussion

### 2.1. The Phylogenetic Analysis of Strain NCPS2016

**Figure 1:**
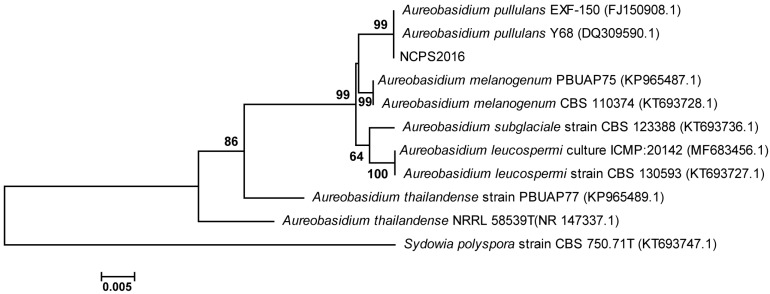
The phylogenetic tree based on the ITS sequences showing the phylogenetic positions of strain NCPS2016, other *Aureobasidium* species, and fungal species relatives. The tree was performed using the neighbor-joining (NJ) methods. Bootstrap values (>50%) based on 1000 replicates are indicated at nodes. Bar 0.005 denotes per nucleotide position.

ITS sequence of strain NCPS2016 was determined and subjected to BLAST searches against the GenBank database (https://blast.ncbi .nlm.nih.gov/Blast.cgi). The phylogenetic tree was constructed with ITS sequences of NCPS2016 and some other type strains in GenBank database. The result showed that ITS sequence of NCPS2016 was similar to those phylogenetically related to *Aureobasidium* spp., and the ITS sequence of NCPS2016 formed a cluster with the most closely related strains *A. pullulans* Y68 and *A. pullulans* EXF-150 (Figure 1). Their similarity was as high as 99%, and NCPS2016 was assigned to the species of *A. pullulans*, named by *A. pullulans* NCPS2016.

### 2.2. Effect of Carbon Source on EPS Production

**Figure 2:**
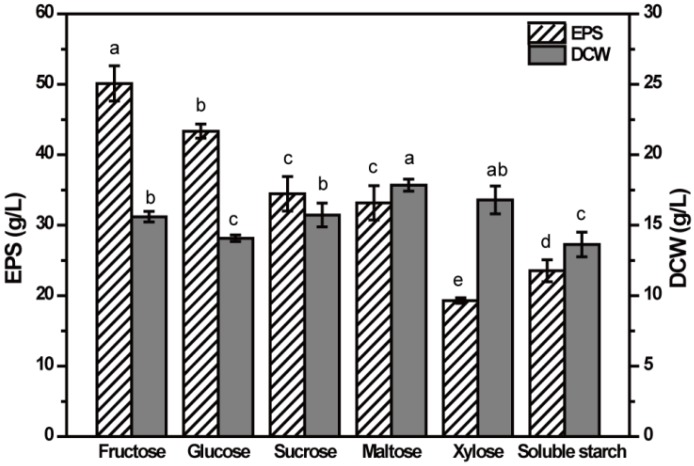
Effect of carbon source on extracellular polysaccharide (EPS) production by NCPS2016. NCPS2016 was cultured in basal medium with carbon source (80 g/L) changed. Data are expressed as means ± SD (*n* = 3). Means with different characters among the same series for different timepoints are significantly different according to Duncan test (*p* < 0.05).

To determine the effect of carbon source on the EPS production, six kinds of sugars of 80 g/L were selected as carbon sources for EPS production by NCPS2016. As shown in Figure 2, the highest EPS production of 50.1 g/L was obtained with fructose as the carbon source, indicating that fructose was the optimal carbon source for the production of EPS of strain NCPS2016, followed by glucose with a production of 43.4 g/L. The EPS production of medium containing sucrose was as high as that of maltose, approximately 34.5 g/L. The results were different from the previously reported stains belonging to *A. pullulans* [14,15,18,19,20], confirming that the best carbon source for EPS production is strain specific. The lowest EPS production was in the xylose fermentation medium, indicating that NCPS2016 showed low ability to use xylose to synthesize EPS, but high ability to use xylose for cellular growth analyzed by dry cell weight (DCW), which was consistent with the earlier studies [17,18]. Maltose was found to be the most favorable carbon source for the growth of NCPS2016, followed by xylose. Also, the dry weight of cells in sucrose and fructose medium was slightly lower. The lowest DCWs were in glucose and soluble starch media. The results indicated that the optimal carbon source for EPS production was not the same as that for the cellular growth. The growth of the strain maintained at a certain level could be beneficial to increase the production of EPS, but it was not exactly that there were more cells for more EPS. The carbon sources showed significantly different influences on EPS production and DCW (*p* < 0.05). Based on the above analysis, fructose was used as the carbon source in subsequent experiments to optimize the medium components for improving the production of EPS.

### 2.3. Effect of SMHs Concentration on EPS Production

**Figure 3:**
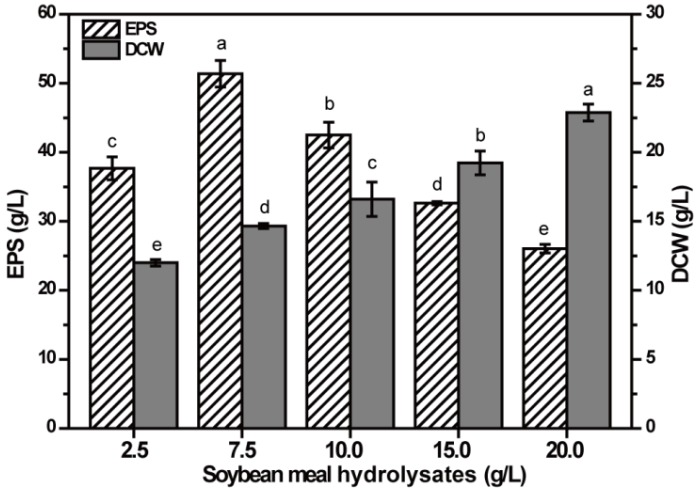
Effect of soybean meal hydrolysate (SMH) contents on the production of EPS by NCPS2016. NCPS2016 was cultured in medium containing 80 g/L fructose with SMH contents changed. The graph shows data from means ± SD (*n* = 3). Means with different characters among the same series for different timepoints are statistical significant according to Duncan test (*p* < 0.05).

Nitrogen is essential for cellular growth and EPS production. Organic nitrogen SMHs were chosen as the nitrogen source with 80 g/L fructose as carbon source for EPS production, and the effect of different concentrations of SMHs on the EPS production was tested (Figure 3). Ranging from 2.5 g/L to 20 g/L SMHs, the EPS production increased first, and then decreased significantly (*p* < 0.05), and the maximum production was obtained in medium containing 7.5 g/L SMHs. In addition, the DCW increased significantly with the increase of SMHs (*p* < 0.05), indicating that the increase in the amount of nitrogen source is conducive to the growth of NCPS2016. However, more nitrogen source could inhibit the synthesis of EPS, consistent with previous report [24]. In order to increase the production of EPS, the amount of nitrogen source should be controlled.

### 2.4. Effects of Inorganic Salts on EPS Production

To investigate the effects of inorganic salts in basal medium on EPS production, differing amounts were added into the media containing 80 g/L fructose as carbon source, and EPS production and DCW were determined.

**Figure 4:**
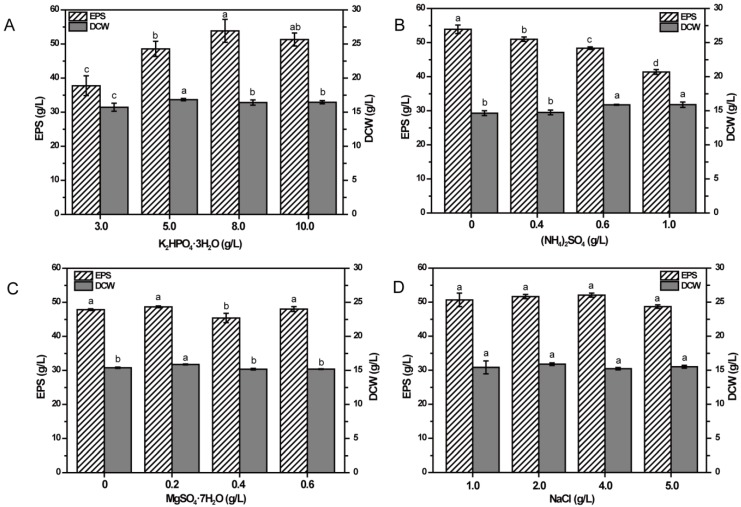
Effects of four kinds of inorganic salts concentrations on EPS production by NCPS2016. The four tested inorganic salts are K_2_HPO_4_·3H_2_O (**A**), (NH_4_)_2_SO_4_ (**B**), MgSO4·7H_2_O (**C**), and NaCl (**D**) in the medium containing 80 g/L fructose. NCPS2016 was cultivated under the conditions as described in Materials and Methods, with the tested single factor being changed. The treatments were performed in triplicate, and data here are from means ± SD. Means with different characters among the same series for different timepoints are significantly different according to Duncan test (*p* < 0.05).

As could be seen in Figure 4A, the production of EPS could be improved significantly (*p* < 0.05) by increasing the concentration of K_2_HPO_4_·3H_2_O until the concentration reached 8.0 g/L, whereas EPS production decreased when the concentration was 10.0 g/L, suggesting that K_2_HPO_4_·3H_2_O might be a key factor on EPS production. Furthermore, DCW also showed a trend of increasing first and then decreasing. When K_2_HPO_4_·3H_2_O was at the concentration of 5.0 g/L, DCW reached the highest level, which was different from the concentration when EPS production reached the highest value, indicating that the lower concentration of K_2_HPO_4_·3H_2_O significantly facilitated the growth of the cells while a higher concentration of K_2_HPO_4_·3H_2_O inhibited the growth of the cells but improved the synthesis of EPS significantly (*p* < 0.05).

Varied concentrations of (NH_4_)_2_SO_4_ ranging from 0–1.0 g/L showed significant effects on EPS production and DCW (*p* < 0.05) (Figure 4B). The highest production of EPS was obtained in the medium without (NH_4_)_2_SO_4_ when the medium contained 10.0 g/L SMHs. The production of EPS decreased with the increase of (NH_4_)_2_SO_4_, while DCW increased with increasing concentration of (NH_4_)_2_SO_4_, which indicated that higher concentration of (NH_4_)_2_SO_4_ could significantly enhance the growth of cells but reduce the production of EPS (*p* < 0.05).

From Figure 4C, the effect of MgSO_4_·7H_2_O was found to be of no significant influence on the production of EPS, but DCW was significantly different, ranging from 0–0.6 g/L (*p* < 0.05). However, 0.4 g/L MgSO_4_·7H_2_O showed significantly less influence on the EPS production than other concentrations (*p* < 0.05). The highest EPS production and DCW were obtained in the medium containing 0.2 g/L MgSO_4_·7H_2_O, so the original concentration of MgSO_4_·7H_2_O in the basal medium of 0.2 g/L was chosen as the concentration in the subsequent experiments.

As shown in Figure 4D, different concentrations of NaCl showed no significant effects on EPS production and DCW (*p* < 0.05). However, at the concentration of 4.0 g/L, the production of EPS was a little higher than that at other concentrations. Therefore, 4.0 g/L NaCl was applied in subsequent experiments.

### 2.5. Optimization of the Medium Components Using Box–Behnken Design (BBD) for EPS Production

Among various methods applied for optimization of medium composition, response surface methodology is the most widely applied method in improving the production of microbial biological macromolecules, such as polysaccharide, glycoprotein, and enzymes [16,25,26]. Based on the single factor experiments, the concentrations of SMHs, (NH_4_)_2_SO_4_ and K_2_HPO_4_·3H_2_O were identified as important variables in influencing EPS production. The three variables were chosen for further optimization with Box–Behnken design ranging from −1 to +1 in relation to EPS production (*Y*). The independent variables SMHs (**A**), (NH_4_)_2_SO_4_ (**B**) and K_2_HPO_4_·3H_2_O (**C**), and their levels, were shown in Table 1. The design matrix and the actual values of EPS production with the predicted values were shown in Table 2. Analysis of the experimental data indicated that the relation between the dependent variable and the independent variables is going to be given by the following second-order polynomial equation:

*Y* (g/L) = 57.83 + 10.13*A* + 1.07*B* + 3.83*C* − 5.09*AB* + 0.8*AC* + 3.51*BC* − 12.37*A*^2^ − 2.64*B*^2^ − 4.51*C*^2^, (1)

The variance analysis using multiple regression was shown in Table 3. The *F*-test analysis was performed to determine the statistical significance of the quadratic model. The “Model *F*-value” of 59.13 implied that the model was significant. Furthermore, the “Lack of Fit *F*-value” of 2.12 implied the lack of fit was not significant relative to the pure error. The CV value was 3.90%, indicating that the experiment has good precision [27]. Furthermore, the fit of the model was checked by the coefficient of determination *R*^2^. The *R*^2^ value of 0.987 indicated that 98.7% of the variability could be explained by the response model. The adjusted determination coefficient *R*^2^ value of 0.9703 was also high enough to indicate the significance of the model. All these parameters were in reasonable agreement between the experimental and the predicted values, and implied that the model could be used to navigate the improvement of EPS production by NCPS2016.

Moreover, the model also suggested that the linear terms of *A* and *C*, the interaction terms of *AB* and *BC*, and the quadratic terms of *A*^2^, *B*^2^ and *C*^2^ were significant in influencing EPS production (*p* < 0.05). In this case, the influence magnitude of the three independent variables on EPS production could be organized by the following order: SMHs (**A**) > K_2_HPO_4_·3H_2_O (**C**) > (NH_4_)_2_SO_4_ (**B**).

**Figure 5:**
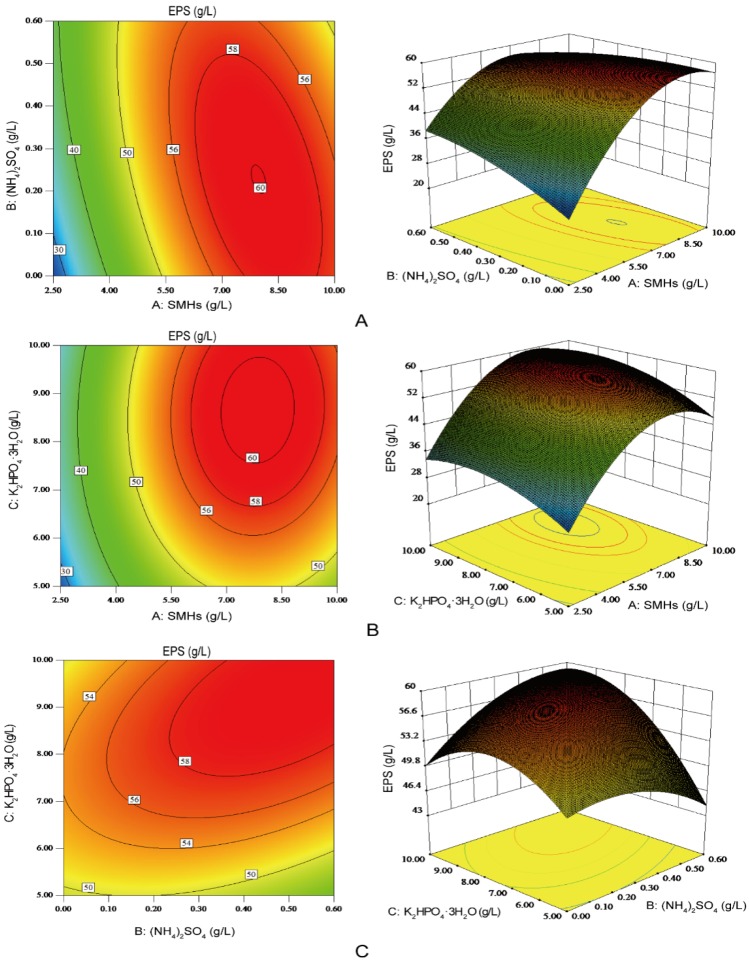
Contour plots (left) and corresponding response surface plots (right) of the effects of three independent variables on the production of EPS. When the effect of two variables was plotted, the other variable was set at the level of zero. (**A**) Interaction between soybean meal hydrolysates (SMHs) and (NH_4_)_2_SO_4_; (**B**) Interaction between SMHs and K_2_HPO_4_·3H_2_O; (**C**) Interaction between (NH_4_)_2_SO_4_ and K_2_HPO_4_·3H_2_O.

The interactions between the any two variables with the third variable maintained at its zero level were depicted by the two-dimensional contour plots and their three-dimensional response surface plots (Figure 5). The elliptical contour plots meant good interactions between the two independent variables [28]. As shown in Figure 5, the interaction between SMHs (**A**) and (NH_4_)_2_SO_4_ (**B**) (Figure 5A), and that between (NH_4_)_2_SO_4_ (**B**) and K_2_HPO_4_·3H_2_O (**C**) (Figure 5C) were significant on EPS production. Furthermore, the interaction between SMHs (**A**) and K_2_HPO_4_·3H_2_O (**C**) was not significant enough to influence EPS production (Figure 5B). By solving the inverse matrix using Design-Expert software, the predicted maximum EPS production of 60.9 g/L could be achieved with the optimal medium composition: SMHs of 7.71 g/L, (NH_4_)_2_SO_4_ of 0.35 g/L, and K_2_HPO_4_·3H_2_O of 8.83 g/L.

### 2.6. Verification of the Optimal Conditions on EPS Production

In order to validate the optimal conditions predicted by the above model, NCPS2016 was cultured in the medium containing 80.0 g/L fructose, 7.71 g/L SMHs, 0.35 g/L (NH_4_)_2_SO_4_, 8.83 g/L K_2_HPO_4_·3H_2_O, 0.2 g/L MgSO_4_·7H_2_O, and 4.0 g/L NaCl. The experiment was performed in triplicate, and EPS production of 59.8 ± 0.6 g/L was obtained, which was considerably similar to the predicted value (60.9 g/L). The perfect agreement between the predicted and the actual values indicated that the model validation and existence of an optimal point.

### 2.7. Pullulanase Hydrolysis Analysis of the Purified EPS

**Figure 6:**
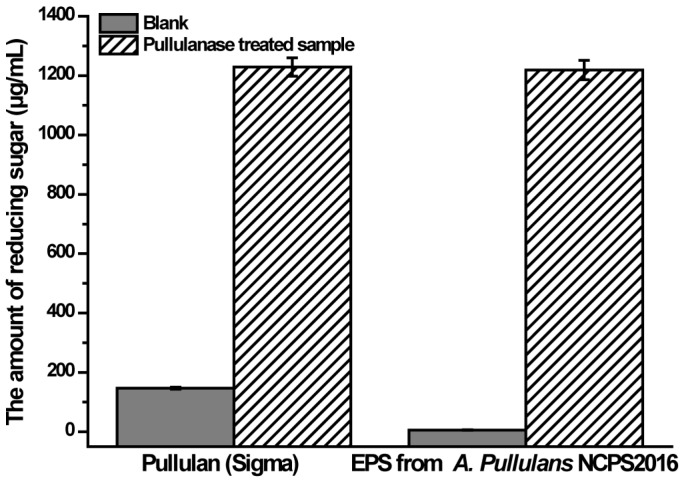
The amounts of reducing sugar in the blank without pullulanase treatment and the sample treated by pullulanase. Data are shown as means ± SD (*n* = 3).

As mentioned previously, pullulan consists of maltotriose repeating units linked with α-1,6 glycosidic linkages [1,2]. In order to verify that the purified EPS from NCPS2016 is pullulan with α-1,6 linkages, the purified EPS was hydrolyzed with pullulanase and the released reducing sugar was determined. The result showed that the reducing sugar of the pullulanase-treated sample increased dramatically compared to that of the untreated blank (Figure 6). That a similar result was observed in the control group of the commercial pullulan indicated that the purified EPS produced by NCPS2016 was pullulan with α-1,6 linkages.

### 2.8. FTIR Spectroscopy of the Purified EPS Produced by NCPS2016

**Figure 7:**
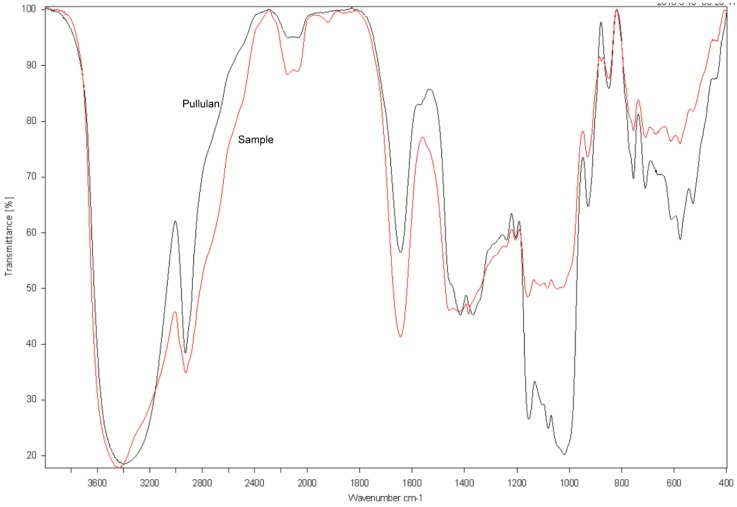
Fourier-transform infrared (FTIR) spectra of the purified EPS (red) by NCPS2016 and standard pullulan (gray) from Sigma (St. Louis, MO, USA).

To further confirm that the purified EPS is pullulan, FTIR spectroscopy was conducted, then the spectrum of the purified EPS was compared with that of the pullulan standard from Sigma. As shown in Figure 7, there was a strong absorption peak at 3340 cm^−1^, which was the stretching absorption peak of the hydroxyl group in the polysaccharide. The absorption peak at 2930 cm^−1^ was the stretching absorption peak of C–H bond on the carbohydrate chain, and the absorption peaks at 1640 cm^−1^ and 1020 cm^−1^ were the stretching absorption peaks of the O–C–O and C–O–C bonds, respectively. Additionally, the absorption peak of α-d glucopyranoside bonds was at 849 cm^−1^, whereas the absorption peaks at 755 cm^−1^ and 915 cm^−1^ indicated that there were α-1,4-d-glucosidic linkages and α-1,6-d-glucosidic linkages, respectively [14,15,29]. These characteristic stretchable peaks of the purified EPS by NCPS2016 were similar to those of the standard pullulan, suggesting that the EPS produced by NCPS2016 is pullulan.

### 2.9. Nuclear Magnetic Resonance (NMR) Spectroscopy of the Purified EPS by NCPS2016

**Figure 8:**
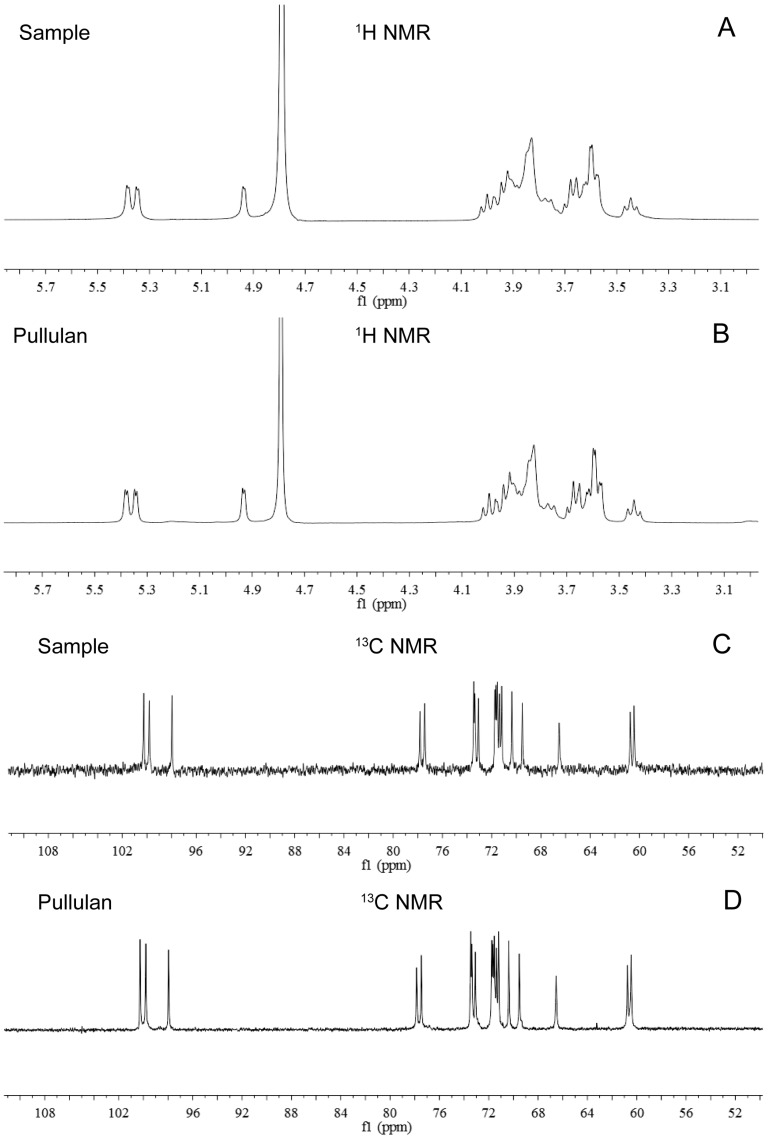
Nuclear magnetic resonance (NMR) spectra of the purified EPS (sample) produced by NCPS2016 and standard pullulan purchased from Sigma. The ^1^H spectra of the purified EPS (sample) and the pullulan standard are shown in (**A** and **B**), respectively, and the displacements of proton peaks of the two appear between *W*_3.3_ and *W*_5.4_. The ^13^C spectra of the purified EPS (sample) and the pullulan standard are shown in (**C** and **D**), respectively, and the displacements of proton peaks of the two appear between *W*_60__.2_ and *W*_101.5_.

Furthermore, the purified EPS produced by NCPS2016 was identified by ^1^H-NMR and ^13^C-NMR. The ^1^H proton peak displacement of the purified EPS by NCPS2016 appeared between *W*_3.3_ and *W*_5.4_ (Figure 8A), while the ^13^C proton peak displcement appeared between *W*_60__.2_ and *W*_101.5_ (Figure 8C). The spectra of ^1^H-NMR and ^13^C-NMR of the pullulan standard from Sigma (St. Louis, MO, USA) were shown in Figure 8B,D, respectively. The pattern of the purified EPS sample was consistent with that of the pullulan standard, which confirmed the identity of the purified EPS produced by NCPS2016 as pullulan [14,30]. Here, combining the results of the pullulanase hydrolysis analysis, FTIR spectrum, and ^1^H-NMR and ^13^C-NMR spectra, a conclusion could be drawn that the EPS produced by NCPS2016 is pullulan.

## 3. Materials and Methods

### 3.1. Strain and Media

*A. pullulans* NCPS2016 was a laboratory-preserved strain isolated from chinar leaves (*Platanus orientalis*). The strain was preserved at −80 °C supplemented with 10% glycerol, and it was cultured on plates containing 20 g/L glucose, 10 g/L yeast extract, 20 g/L peptone, and 15 g/L agar at 28 °C, then maintained at 4 °C for short-time use.

The seed medium containing 20 g/L glucose, 2.5 g/L yeast extract, 0.6 g/L (NH_4_)_2_SO_4_, 0.2 g/L MgSO_4_·7H_2_O, 1.0 g/L NaCl, and 6.6 g/L K_2_HPO_4_·3H_2_O (pH 6.5) was sterilized at 115 °C for 30 min.

The SMHs were prepared by acidolysis of soybean meal with 0.25 M HCl at 121 °C for 30 min. Then, the cooled hydrolysates solution was neutralized by 1 M NaOH. The basal medium for EPS fermentation containing 80 g/L glucose, 10 g/L SMHs, 0.2 g/L MgSO_4_·7H_2_O, 4.0 g/L NaCl, 0.6 g/L (NH_4_)_2_SO_4_, and 5 g/L K_2_HPO_4_·3H_2_O (pH 7.0) was sterilized at 115 °C for 30 min. All the chemicals (Sinopharm Chemical Reagent Co., Ltd, Shanghai, China) were of analytical reagent grade except soybean meal, while soybean meal was purchased from the feed market (Jinan, China) and was the same batch of products.

### 3.2. Inoculum Preparation and Flask Fermentation

For inoculum preparation, NCPS2016 was cultured on the plate at 28 °C for 2 days, and 2-ring slant inoculum was taken into the seed medium and cultivated at 28 °C, 220 rpm for 24 h. For fermentation, 5% (*v*/*v*) inoculum was transferred to the fermentation medium, and cultivated at 28 °C, 220 rpm for 84 h.

### 3.3. Preparation of Crude EPS and Determination of Dry Weight of Cells (DCW)

The fermentation broth was centrifuged in a refrigerated centrifuge at 10,000 rpm at 4 °C for 20 min. The supernatant was collected, and the EPS was precipitated with 2 volumes of pre-chilled 95% ethanol, and then the precipitate was washed twice with pre-chilled 95% ethanol. It was dried at 80 °C until constant weight. The pellets after centrifugation of the fermentation broth were washed with distilled water three times. The cells were placed in an oven at 80 °C and dried to a constant weight. Then they were collected and weighed.

### 3.4. Effect of Varied Carbon Sources on the Production of EPS

Five different sugars of 80 g/L (sucrose, fructose, maltose, xylose, and soluble starch) were used as carbon sources to replace glucose in the basal fermentation medium for EPS production of NCPS2016 under the condition described above, and the production of EPS and DCW were measured. All the experiments were performed in triplicate and the results were shown as means ± standard deviation (SD).

### 3.5. Optimization by Single-Factor Test for EPS Production

Using 80 g/L fructose as the carbon source, five single factors were investigated for their effects on the production of EPS, including different concentrations of SMHs (2.5 g/L, 7.5 g/L, 10.0 g/L, 15.0 g/L, and 20.0 g/L) as the nitrogen source, different concentrations of K_2_HPO_4_·3H_2_O (3.0 g/L, 5.0 g/L, 8.0 g/L, and 10.0 g/L), varied concentrations of (NH_4_)_2_SO_4_ (0, 0.4 g/L, 0.6 g/L, and 1.0 g/L), a series of concentrations of MgSO_4_·7H_2_O (0 g/L, 0.2 g/L, 0.4 g/L, and 0.6 g/L) and different concentrations of NaCl (1.0 g/L, 2.0 g/L, 4.0 g/L, and 5.0 g/L). NCPS2016 was cultivated under the fermentation condition described above with a change of the tested single factor, and the production of EPS and DCW were determined. All the measurements were conducted in triplicate and the results were expressed as means ± SD.

### 3.6. Optimization by RSM for EPS Production

Based on the results of single-factor test, the concentrations of SMHs, (NH_4_)_2_SO_4_, and K_2_HPO_4_·3H_2_O as independent variables were further optimized by BBD with three-factor and three-level using Design-Expert software [27]. Seventeen experimental runs were conducted in triplicate, and the mean value of the production of EPS was taken as response value. The independent variables and the levels were shown in Table 1. The contents of NaCl and MgSO_4_·7H_2_O were 4 g/L and 0.2 g/L in the medium, respectively. 

### 3.7. Statistical Analysis

The results were described as means with standard deviations. One-way analysis of variance (ANOVA) was applied to analyze the data. The means were compared using Duncan test at *p* < 0.05. Data analyses were performed with a trial version of IBM SPSS Statistics 22 software (IBM Corp., Armonk, New York, NY, USA).

The results of BBD were described as the following second-order polynomial regression model:

Y = β_0_+ ∑β_i_X_i_ + ∑β_ij_X_i_X_j_ + ∑β_ii_X_i_^2^, (2)

where Y represented the predicted response (the production of EPS in g/L), and β_0_, β_i_, β_ij_, and β_ii_ denoted regression coefficients for the intercept, the linear coefficient, the interactive coefficient, and the quadratic coefficient, respectively, while X_i_ and X_j_ representing the coded values of the independent variables [25]. The results were analyzed by ANOVA, and the interactions between the independent variables were revealed by 2-D contour plots and 3-D response surface using a trial version of Design-Expert software (version 8.0.4, Stat-Ease Inc., Minneapolis, MN, USA).

### 3.8. Purification of Crude EPS

The crude EPS was purified according to the previously reported method with slight modification. The crudeEPS was dissolved with distilled water at a concentration of 5%. The solution was washed using *N*-butanol/trichloromethane to remove proteins. After the protein was removed, the sample was dialyzed against distilled water for two days to remove small molecules, then the purified EPS was lyophilized to obtain the pure EPS powder [14].

### 3.9. Hydrolysis Analysis of the Purified EPS

The purified EPS or the standard pullulan (Sigma-Aldrich Corp., St. Louis, MO, USA) of 0.1 g was dissolved in 10 mL distilled water at 80 °C. EPS solution of 1 mL was mixed with 0.9 mL of 50 mM sodium acetate buffer (pH 4.5) and 0.1 mL pullulanase, which was purchased from Sigma. Then, the mixture was incubated at 60 °C for 15 min in a water bath, and the reducing sugar released was detected using the Nelson–Somogyi method [29].

### 3.10. Fourier-Transform Infrared (FTIR) Spectroscopy of the Purified EPS

The purified EPS was characterized using a Nicolet Nexus FTIR 470 spectrometer (Thermo Electron Corporation, Waltham, MA, USA). The prepared EPS sample of 2 mg was mixed with 60 mg of potassium bromide, and the sample was performed over a range of 4000–400 cm^−1^ at a rate of 16 scans with a resolution of 2 cm^−1^. The standard pullulan (Sigma-Aldrich Corp., St. Louis, MO, USA) was performed under the same condition [16,29].

### 3.11. Nuclear Magnetic Resonance (NMR) Spectroscopy of the Purified EPS

The purified EPS was recorded on a Bruker NMR spectrometer (Karlsruhe, Germany). The sample of 50 mg was dissolved in 500 μL D_2_O, and the ^1^H-NMR spectrum was acquired with a spectral width of 8000 Hz, pulse width of 11.8 μs, acquisition time of 4 s, accumulation of 16 times. The ^1^H spectrum was collected at a frequency of 400 MHz, while the ^13^C-NMR spectrum was collected at a frequency of 100 MHz. The ^13^C-NMR spectrum was obtained with a spectral width of 26,000 Hz, with 12.2 μs pulse width, acquisition time of 4 s, and 12,000 scans. All the measurements were performed at 20 °C.

### 3.12. DNA Extraction, PCR Amplification, ITS Sequencing, and Phylogenetic Analysis

The ITS sequences of different species are highly conserved, and are commonly used for molecular identification of eukaryotic microorganisms [29]. The genome DNA of NCPS2016 was extracted according to the instructions of the fungal genome extraction kit (Shanghai Shenggong, Shanghai, China), and the conserved spacer sequence ITS was amplified using the ITS universal primers ITS1: TCCGTAGGTGAACCTGCGG and ITS4: TCCTCCGCTTATTGATATGC. The ITS sequence was sequenced and BLAST alignment was performed after sequencing (https://blast.ncbi .nlm.nih.gov/Blast.cgi). Multiple alignments were conducted using MEGA6 to perform evolutionary relationship analysis by neighbor-joining method [31].

### 3.13. Nucleotide Sequence Accession Number

The ITS sequence for *A. pullulans* NCPS2016 was deposited in GenBank under the accession number MH202939.

## 4. Conclusions

In this study, a pullulan-producing strain was confirmed by morphological analysis (data not shown) and phylogenetic analysis, and it was named by *A. pullulans* NCPS2016. Here, the single-factor test and the BBD are found to be efficient methods to improve the production of EPS produced by NCPS2016. The maximum EPS production was obtained when NCPS2016 was cultivated in the optimal medium containing 80.0 g/L fructose, 7.71 g/L SMHs, 0.35 g/L (NH_4_)_2_SO_4_, 8.83 g/L K_2_HPO_4_·3H_2_O, 0.2 g/L MgSO_4_·7H_2_O and 4.0 g/L NaCl with initial pH value of 7.0. The production of pullulan reached 59.8 ± 0.6 g/L in flask fermentation in the optimal medium, which was approximately 39% higher than that before optimization (43.4 ± 1.0 g/L). The model was validated by experimental performance, and the actual production of pullulan was close to the predicted value of the model, indicating that the established model in this report is efficient to navigate the production of pullulan by NCPS2016. The improvement of pullulan production is beneficial to reduce its production cost. The results here provide a basis for further industrial-scale production of pullulan.

In addition, the optimal carbon source for NCPS2016 to produce pullulan is fructose, which is different from many previous observations reporting that the optimal carbon source for pullulan production by *A. pullulans* is sucrose [17,18,19] or glucose [14,20]. This suggests that the best substrate for pullulan synthesis is strain specific, and the mechanism for NCPS2016 utilizing fructose efficiently to synthesize pullulan is worthy of further investigation. In the near future, the effect of fructose on the expression of key enzymes (α-phosphoglucomutase, UDPG-pyrophosphorylase and glucosyltransferase) for pullulan synthesis should be analyzed according to the methods of Duan and Wang [19,20]. In the future, the transcriptomes of NCPS2016 cultured in fructose, glucose, and sucrose medium could be determined, respectively. Furthermore, the genes of differential expression should be classified, especially the genes involved in carbon metabolism and pullulan synthesis. The comparative transcriptome combined with the molecular genetics methods might be applied to illustrate the specific mechanism for pullulan production of NCPS2016.

## Figures and Tables

**Table 1:** Variables and levels in Box–Behnken design of fermentation medium components for EPS production.

Level	*A*: SMHs (g/L)	*B*: (NH_4_)_2_SO_4_ (g/L)	*C*: K_2_HPO_4_·3H_2_O (g/L)
−1	2.50	0	5.00
0	6.25	0.30	7.50
1	10.00	0.60	10.00

**Table 2:** The matrix of Box–Behnken design and the corresponding experimental and predicted values of EPS production of strain NCPS2016.

Run	*A*	*B*	*C*	EPS (g/L)	
				Predicted Value	Actual Value
1	−1	−1	0	26.54	24.93
2	0	0	0	57.83	57.55
3	1	1	0	48.94	50.55
4	0	0	0	57.83	55.23
5	0	−1	−1	49.29	49.48
6	1	0	1	55.72	54.29
7	0	−1	1	49.94	51.66
8	1	0	−1	46.45	46.56
9	0	1	1	59.1	58.91
10	1	−1	0	56.98	56.68
11	−1	0	−1	27.78	29.21
12	0	1	−1	44.4	42.68
13	0	0	0	57.83	59.03
14	0	0	0	57.83	58.78
15	−1	0	1	33.86	33.75
16	0	0	0	57.83	58.56
17	−1	1	0	38.84	39.14

**Table 3:** Variance analysis of response surface quadratic model for EPS production of strain NCPS2016.

Source	*df*	EPS Production ^a^			
		Sum of Squares	Mean Square	*F*-Value	*p*-Value Prob > *F*
Model	9	1913.83	212.65	59.13	<0.0001 *
*A*	1	821.14	821.14	228.32	<0.0001 *
*B*	1	9.1	9.1	2.53	0.1558
*C*	1	117.66	117.66	32.72	0.0007 *
*AB*	1	103.43	103.43	28.76	0.001 *
*AC*	1	2.54	2.54	0.71	0.4281
*BC*	1	49.35	49.35	13.72	0.0076 *
*A* ^2^	1	644.02	644.02	179.07	<0.0001 *
*B* ^2^	1	29.29	29.29	8.14	0.0246 *
*C* ^2^	1	85.64	85.64	23.81	0.0018 *
Residual	7	25.17	3.6		
Lack of Fit	3	15.46	5.15	2.12	0.2401
Pure Error	4	9.71	2.43		
Cor Total	16	1939			

* Model terms are significant (*p* < 0.05). ^a^*R*^2^ = 0.987; Adj *R*^2^ = 0.973; CV = 3.90%.
